# Reported disability in relation to observed activity limitation, grip strength and physical function in women and men with rheumatoid arthritis

**DOI:** 10.1186/s41927-021-00184-5

**Published:** 2021-05-03

**Authors:** Sidona-Valentina Bala, Maria L. E. Andersson, Kristina Forslind, Björn Svensson, Ingiäld Hafström

**Affiliations:** 1 Department of Health Sciences, Faculty of Medicine, Lund University, Lund, Sweden; 2 Department of Medicine, Section of Rheumatology, Helsingborg’s Hospital, Helsingborgs lasarett, Olympiahuset plan 2, S-251 87 Helsingborg, Sweden; 3 Department of Rheumatology, Faculty of Medicine, Lund University, Lund, Sweden; 4 Spenshult Research and Development Center, Halmstad, Sweden; 5 Department of Research and Education, Skånevård Sund, Region Skåne, and Helsingborg’s Hospital, Helsingborg, Sweden; 6 Department of Medicine, Division of Gastroenterology and Rheumatology, Karolinska Institutet, Huddinge, Stockholm, Sweden; 7 Rheumatology Unit, Karolinska University Hospital, Stockholm, Sweden

**Keywords:** Rheumatoid arthritis, Disability, HAQ, Mobility, Hand strength, Women, Men

## Abstract

**Background:**

The self-reported Health Assessment Questionnaire (HAQ) is specifically designed to assess disability in arthritic patients. In many studies women report higher functional disability than men. The reasons for this difference are suggested to be multifactorial. We therefore evaluated functional disability assessed by HAQ in women and men with rheumatoid arthritis (RA) in relation to observed disability, grip force and physical function.

**Methods:**

Patients with RA, 51 women and 49 men, completed the HAQ on three occasions, some weeks apart. Between HAQ1 and HAQ2, all patients performed 17 of the 20 activities (7 domains) included in the HAQ under observation in a specially designed environment, the observed HAQ. During the same day, grip force, measured by GRIPPIT and physical function assessed by the SOFI (Signals of Functional Impairment) index were evaluated. Differences between groups were studied by the chi-square test, Mann-Whitney U test and Wilcoxon Sign Rank test. Correlations were analysed by Spearman rank correlation. Comparisons between repeated measures were performed using Friedman’s test.

**Results:**

Median (IQR) total HAQ1 score was 0.50 (0.88) for women and 0.25 (0.84) for men, *p* = 0.038, and the observed HAQ score (7 domains) 0.57 (0.9) for women and 0.43 (0.96) for men, *p* = 0.292. The correlations between reported HAQ1 score (7 domains) and observed HAQ score were strong, *r* = 0.860, *p* < 0.001 in women, and *r* = 0.820, *p* < 0.001 in men. For some activities the patients, both women and men, reported lower difficulty than that observed. Women had lower grip force than men, median (IQR), right and left 126 (84) Newton, versus 238 (146), *p* < 0.001, and there was a negative correlation between grip force and most of the separate activities in HAQ in both genders. SOFI index was similar in women and men, median (IQR) 0 (3.0) versus 0 (2.0), *p* = 0.277, with a moderate correlation to HAQ.

**Conclusions:**

The results indicate that in well-treated patients with RA the correlations between reported and observed HAQ scores were strong, similarly in women and men. We found no evidence that the patient’s opinion was dependent on unawareness of her/his own ability.

**Supplementary Information:**

The online version contains supplementary material available at 10.1186/s41927-021-00184-5.

## Background

In the last decades, reports have emerged that women with rheumatoid arthritis (RA) have a more severe disease course than men [1–6]. In both genders, disease activity decreases over the first 8 years from disease onset but more in men, whereas functional disability, after an initial decrease, levels off at a higher level in women than in men [7, 8]. After 5 and 8 years from disease onset, women have higher disease activity as measured by the Disease Activity Score 28 (DAS28) and higher functional disability measured by Health Assessment Questionnaire (HAQ) than men [7–9]. These differences between genders contrast with the fact that joint destruction in hands and feet was similar in women and men, both after 5 and 8 years from disease onset [5, 6]. The reasons for these gender differences have not yet been identified. As to functional disability, potential causes may be that women’s grip force is lower [10], that men overestimate their functional ability [5, 11], that men have better muscle strength [12] and /or that women report more pain [13–15]. Another reason may be that HAQ is not sufficiently developed with regard to possible gender differences and therefore cannot handle functional differences between the sexes. This is of great importance to be clarified as HAQ is the most widely used instrument to assess disability in studies of RA. Moreover, HAQ has proved to be a predictor of disability [16] and sustained remission in the course of RA [17], and also a decision support for need of multidisciplinary interventions [18]. Therefore, additional knowledge about the reasons for the differences between women’s and men’s subjective assessments of their functional disability is necessary.

The aims of the present study were to evaluate functional disability assessed by HAQ in women and men with RA in relation to observed disability, grip force and physical function.

## Methods

### Design and setting

This is a cross-sectional study performed in an outpatient rheumatology clinic located at a central hospital in Southern Sweden.

### Patients

In all, 100 patients fulfilling the classification criteria for RA established by the American Rheumatism Association [19] were included in the study. The women and men were separately consecutively included with the aim to achieve about equal numbers. The patients were eligible for inclusion if they were 18–74 years of age, had a disease duration less than 15 years and a good understanding of the Swedish language. They were asked for participation in the study in connection with their regular visits to a nurse. Nine patients were not asked to participate - 4 were wheelchair users (3 women and 1 man) and 5 had undergone hip surgery (1 woman and 4 men) - as their functional limitations might have affected their ability to perform some of the activities in the study protocol. Fifteen patients (6 women and 9 men) declined participation because they did not have any opportunities to return to the clinic within 2 weeks according to stage 2 in the study protocol. All included patients completed the study.

### Assessments

#### Health assessment questionnaire (HAQ)

Functional disability was assessed by the Swedish version of the HAQ [20]. The HAQ comprises of 20 questions covering 20 daily activities. These are divided in eight domains: dressing and grooming, rising, eating, walking, hygiene, reaching, gripping, and other activities. Each question is scored according to a four-point scale (0–3), and the highest scores from each domain are summed and divided by eight, to derive a total HAQ score, which ranges from 0 to 3 (3 = highest level of disability).

#### Disease activity score 28 (DAS28)

Disease activity was assessed by the composite index Disease Activity Score calculated in 28 joints (DAS28; range 0–9.4, best to worse) [21].

#### Grip force

Grip force was assessed in both hands by the electronic instrument GRIPPIT, which measures grip force in newtons (N) [22]. Average values over a 10 s uninterrupted grip were recorded. Results are given for the separate hands as well as means for right and left hands.

#### Physical function

Physical function was assessed by the SOFI (Signals Of Functional Impairment) index which is a three parts measurement of hands (range 0–16), upper extremities/arms (range 0–12), and lower extremities/legs (range 0–16) [23]. The SOFI index ranges from 0 (best) to 44 (worst).

### Data collection procedure

Data were collected at baseline (stage 1), within 2 weeks after baseline (stage 2), and within 3 weeks after stage 2 (stage 3).

#### Stage 1

At a regular visit to the nurse, all patients were asked to complete a HAQ, hereinafter referred to as HAQ1. Demographic data and their last DAS28 (values not older than 6 month) were also recorded. The patients were then planned for a new visit to the nurse within 2 weeks.

#### Stage 2

At a second visit all patients performed 17 of the 20 activities included in the HAQ in a specially designed environment within the hospital in order to compare their subjective assessments with their observed functional abilities. These 17 activities corresponded to 7 of the 8 domains in the questionnaire. Exceptions were made for the activities in the first domain (hair washing and dressing, corresponding to questions 1 and 2 in the HAQ) and for one activity in the eighth domain (common activities, corresponding to the question “Can you handle your own household purchases?”) because these activities were difficult to perform in hospital environment.

During this assessment procedure, which lasted for 45 min (min) to 65 min (max), the patient was assisted by a nurse who observed and estimated the actual functional performance. The nurse documented her assessment in the HAQ questionnaire (hereinafter referred to as observed HAQ), without any feedback to the patient of how her/his performance was assessed. To facilitate and ensure consistency in the nurse’s assessment, a standardized protocol which described the different levels of ability for the HAQ-scale’s response categories “With some difficulty” and “With much difficulty” has been developed by using expert opinion (rheumatologists, occupational therapist, nurse and health care researchers) (Table 1). The protocol was tested on 4 patients, 2 men and 2 women, which did not result in any adjustments. According to the protocol the time factor was not considered as an assessment criterion.

**Table 1:** Standardized assessment protocol for Health Assessment Questionnaire response categories “With some difficulty” and “With much difficulty”

*Activity/Question*	*With some difficulty*	*With much difficulty*
3. Are you able to stand up from a chair without support?	a. With the support of one hand against the chair	a. With the support of both hands against the chair
	b. Succeed on the second attempt	b. Uncertainty / fear exists
		c. Succeeds only on the third attempt or more
4. Are you able to get in and out of bed?	a. Uses own technique of raising	a. Needs to take support in some way
	b. Succeed on the second attempt	b. Must use a different technique than usual
		c. Succeeds only on the third attempt or more
5. Are you able to cut your own meat? *The meat is replaced with half an orange. Ordinary knife is used*	a. A little clumsiness	a. Greater clumsiness, dropping of utensils
	b. Somewhat difficult with the grip	b. Uncoordinated cutting
	c. Succeed on the second attempt	c. Very difficult to grip
		d. Succeeds only on the third attempt or more
6. Can you cook your own food? *Patients prepare a soup according to recipe. Normal cooking time 10 min*	a. A little clumsiness	a. Greater clumsiness, drops the utensils
	b. A bit difficult with grip and handling of pans and utensils	b. Difficult to open, to lift, must exert oneself
	c. Hard to open a bottle, succeeds on the second try	c. Succeeds first on third attempt or more
7. Are you able to lift a full cup or glass to your mouth?	a. A little clumsiness	a. Overflows
	b. Uses an unusual grip	b. Uses both hands
8. Are you able to walk five steps down a stairway?	a. Little uncertainty exists	a. Holds on to the railing
	b. Watches carefully when taking a step	b. Takes one step at a time
		c. Is very uncertain/afraid
9. Are you able to walk outdoors on flat ground?	a. Pays close attention to the ground while walking	a. Very unstable
	b. A little unsteady	b. Not able to walk more than 15–20 m
	c. Walks with caution	c. Fear of falling exists
10. Are you able to take a bath? *Simulated in bathtub*	a. A bit difficult to take the step over, succeeds on the second attempt	a. Succeeds on the third attempt to take the step over
	b. Careful when he / she sits down, supports with both hands	b. Supporting oneself with both hands on the bathtub edge to get in
	c. Little uncertainty	c. Very difficult to get up, succeeds on the third attempt
	d. Manages to get up on the second attempt	d. Very uncertain/afraid
11. Are you able to get on and off the toilet?	a. A little cautious, looking for some support with one hand	a. Takes support with both hands
	b. Manages to get up on the second attempt	b. Sits uncontrollably
		c. Manages to get up on the third attempt
12. Are you able to wash and dry your body? *Simulated*	a. A little clumsiness with soap and shampoo	a. Does not reach multiple body parts, > 2
	b. Not able to reach everywhere (not back and feet) but almost	b. Very difficult with the balance when drying
		c. Needs to sit
13. Are you able to take a 2-k bag of sugar of a shelf at head height?	a. Takes help of both hands	a. Risk of dropping the bag due to weak grip
	b. Uses an unusual grip	b. Succeeds on the third attempt or more
	c. Some effort succeeds on the second attempt	
14. Are you able to bend over to pick up clothing from the floor?	a. Some effort	a. Losing the grip
	b. Little clumsiness	b. Risk of fall
	c. Succeed on the second attempt	c. Succeeds on the third attempt or more
15. Are you able to open car doors?	a. Difficulty with the grip, losing the grip, succeeding on the second attempt	a. Very heavy, using both hands
	b. Little difficult, it’s heavy	b. Succeeds on the third attempt
16. Are you able to open previously opened jars?	a. Not directly, succeeds on the second attempt	a. Greater clumsiness
	b. Little clumsiness	b. Uses both hands
	c. Takes an unusual technique (for example holding the jar against the body)	c. Risk of dropping the jar due to the grip
17. Are you able to turn a water tap on and off?	a. Uses an unusual grip	a. Uses both hands
	b. Losing the grip	b. Succeeds on the third attempt or more
	c. Succeeds on the second attempt	
18. Are you able to vacuum? *Tested on delimited surface, same for all patients*	a. Weak grip	a. Loses the grip
	b. Somewhat difficult to pull the vacuum cleaner	b. Very difficult to pull the vacuum cleaner
	c. Not able to reach under low furniture	c. Takes short breaks
	d. Uses an unusual technique (e.g. hose around waist)	d. Not able to reach everywhere on free surfaces
20. Are you able to get in and out of a car?	a. Uses an unusual technique	a. Sits uncontrollably
	b. Some risk of hitting the head	b. Losing balance
	c. Succeeds on the second attempt	c. Risk of falling
		d. Great risk of hitting the head
		e. Succeeds on the third attempt or more when raised

After performing the observed activities, the patients were asked to complete the HAQ questionnaire once again (hereinafter referred to as HAQ2).

At this time point the occupational therapist assessed the patients’ physical function in the upper and lower extremities by SOFI, and their grip force for both hands by GRIPPIT.

#### Stage 3

One to 3 weeks after stage 2, the patients were asked to complete a final HAQ questionnaire (HAQ3). The HAQ3 questionnaire was marked with date and sent to the patients by post together with a response envelope. Patients who did not return the questionnaire within 2 weeks were reminded by telephone.

### Statistical analysis

Demographics and disease activity data were analysed descriptively. To study the differences between groups, the chi-square test, Mann-Whitney U test and Wilcoxon Sign Rank test were used when appropriate. Correlations were analysed by Spearman rank correlation. Comparisons between repeated measures were performed using Friedman’s test. Group comparisons were conducted on HAQ- questions and HAQ-total scores. The observed HAQ score was calculated on 7 domains. The sum of the highest scores in each domain was divided by seven to achieve a total HAQ score. When comparing observed HAQ with reported HAQ, the same 17 questions were used, and a total score calculated. The number of included patients, 51 women and 49 men, was considered adequate, based on a study showing that a change in HAQ of 0.31 points or more over 2 months was needed to ensure a clinically relevant change with a significance level of 5% and a statistical power of 80% [24].

All statistical analyses were two-tailed and performed using IBM SPSS version 21(IBM Corp., Armonk, NY, USA). For all tests *p* < 0.05 was considered to be significant.

## Results

### Demographic and clinical characteristics

A total of 100 patients, 51 women and 49 men were included in the study. Table 2 shows their characteristics. Both genders had low disease activity, the women were younger, had higher HAQ and had lower grip force than the men. All patients were treated with conventional or biological disease-modifying anti-rheumatic drugs.

**Table 2:** Characteristics of the patients split by gender

	Women *n* = 51	Men *n* = 49	*p*-value
Age (years)	58 (20)	66 (14)	0.008
Disease duration (years)	5 (8)	5 (9)	0.590
RF positive, n (%)	44 (86)	34 (69)	0.042
DAS28	2.65 (1.31)	2.52 (1.55)	0.450
HAQ1	0.50 (0.88)	0.25 (0.84)	0.038
Grip force right, N	117 (89)	241 (158)	< 0.001
Grip force left, N	104 (105)	245 (131)	< 0.001
Grip force mean right + left, N	126 (84)	238 (146)	< 0.001
SOFI hands	1.0 (2.0)	1.0 (3.0)	0.663
SOFI arms	0 (1.0)	0 (2.0)	0.303
SOFI legs	1.9 (3.0)	1.3 (2.7)	0.283
SOFI index	0 (3.0)	0 (2.0)	0.277
cDMARDs, n (%)	24 (47)	31 (63)	0.103
bDMARDs, n (%)	27 (53)	18 (37)	

Values are median (IQR), or numbers (%); *p*-values are differences between women and men

*RF* rheumatoid factor, *n* numbers, *DAS28* disease activity score calculated on 28 joints, *HAQ1* Health Assessment Questionnaire reported at baseline, *N* Newton, *SOFI* Signals of Functional Impairment, *cDMARDs* conventional disease-modifying anti-rheumatic drugs, *bDMARDs* biological disease-modifying anti-rheumatic drugs

### Comparisons between reported and observed HAQ

Both women and men reported in HAQ1 that their abilities to perform the activities regarding cooking your own food (question 6) and washing and drying yourself (question 12), were better than what was later observed (Table 3).

**Table 3:** Reported and observed HAQ in women and men. The scores for the 17 questions in reported HAQ1 and observed HAQ are compared, as well as the total HAQ scores for all 7 domains

	Women			Men		
	HAQ1 reported	HAQ observed	*p*-value	HAQ1 reported	HAQ observed	*p*-value
Rising						
HAQ_3	0.0 (0.8)	0.0 (0.0)	0.096	0.0 (1.0)	0.0 (1.0)	0.180
HAQ_4	0.0 (0.0)	0.0 (1.0)	0.180	0.0 (1.0)	0.0 (0.0)	0.157
Eating						
HAQ_5	0.0 (1.0)	0.0 (1.0)	0.248	0.0 (0.0)	0.0 (1.0)	0.058
HAQ_6	0.0 (0.8)	0.0 (1.0)	**0.003**	0.0 (0.0)	0.0 (1.0)	**0.013**
HAQ_7	0.0 (0.0)	0.0 (0.0)	0.180	0.0 (0.0)	0.0 (0.0)	1.00
Walking						
HAQ_8	0.0 (1.0)	0.0 (1.0)	0.206	0.0 (0.0)	0.0 (0.8)	0.052
HAQ_9	0.0 (1.0)	0.0 (1.0)	1.00	0.0 (0.0))	0.0 (1.0)	0.071
Hygiene						
HAQ_10	0.0 (1.0)	0.0 (2.0)	**0.015**	0.0 (1.0)	0.5 (1.8)	0.058
HAQ_11	0.0 (0.0)	0.0 (0.0)	0.317	0.0 (0.0)	0.0 (1.0)	0.317
HAQ_12	0.0 (1.0)	0.0 (1.0)	**0.029**	0.0 (1.0)	1.0 (1.0)	**< 0.001**
Reaching						
HAQ_13	1.0 (1.0)	1.0 (1.0)	1.00	0.0 (1.0)	0.0 (1.0)	**0.035**
HAQ_14	0.0 (0.0)	0.0 (0.5)	1.00	0.0 (1.0)	0.0 (1.0)	0.180
Gripping						
HAQ_15	0.0 (0.0)	0.0 (0.0)	0.564	0.0 (0.0)	0.0 (0.0)	0.317
HAQ_16	0.0 (1.0)	0.0 (1.0)	0.071	0.0 (1.0)	0.0 (1.0)	**0.046**
HAQ_17	0.0 (1.0)	0.0 (0.5)	0.180	0.0 (0.0)	0.0 (0.0)	0.157
Other activities						
HAQ_18	0.0 (1.0)	0.0 (1.0)	0.096	0.0 (0.0)	0.0 (1.0)	**0.002**
HAQ_20	0.0 (1.0)	0.0 (1.0)	0.366	0.0 (1.0)	0.0 (1.0)	0.564
All 7 domains						
HAQ_score	0.50 (1.0)	0.57 (0.9)	0.325	0.28 (0.9)	0.43 (0.96)	**0.002**

Values are median (IQR); *p*-values are differences between reported and observed HAQ in respective gender. Bold *p*-values are significant

*HAQ* Health Assessment Questionnaire

In addition, women reported their ability to be better than was observed also regarding taking a bath in a bathtub (question 10) and men regarding taking a package of sugar from a shelf (question 13), opening cans with screw cap (question 16) and being able to vacuum (question 18) (Table 3).

In women, the reported HAQ1 score (7 domains) did not significantly differ from the corresponding observed HAQ score, while in men, the reported HAQ1 score was significantly lower than that observed, median (IQR) 0.28 (0.9) versus 0.43 (0.96), *p* = 0.002 (Table 3).

Despite the differences in some of the activities, the correlations between reported HAQ1 score (7 domains) and observed HAQ score were strong, *r* = 0.860, *p* < 0.001 in women, and *r* = 0.820, *p* < 0.001 in men.

We then compared the observed HAQ with that reported by the patients after the supervised performance of the different activities, HAQ2. The pattern of differences in the respective activity was very similar to that found when comparing observed HAQ with HAQ1 (Table S1). Reported HAQ2 score (7 domains) and observed HAQ score were in women median (IQR) 0.43 (0.96) and 0.57 (0.86), respectively, *p* = 0.002, and in men 0.21 (0.57) and 0.43 (0.96), *p* < 0.001. The correlations between reported HAQ2 score (7 domains) and observed HAQ score was still strong, *r* = 0.961, *p* < 0.001 in women, and *r* = 0.942, *p* < 0.001, in men.

### Observed HAQ in women and men

When comparing how women and men performed the observed HAQ activities, it was found that in only two of the 17 items women performed worse than men, namely the question about rising from a chair (question 5), median (IQR) 0.0 (1.0) vs 0.0 (1.0), *p* = 0.023 and reaching (number 13), 1.0 (1.0) vs 0.0 (1.0), *p* = 0.014. The observed HAQ score (7 domains) did not differ between genders, median (IQR) 0.57 (0.9) versus 0.43 (0.96), *p* = 0.292.

### Repeated HAQ measurements

We further analysed if the reported HAQ was influenced by the patient’s experience of the observed HAQ performance by comparisons of HAQ1, HAQ2 and HAQ3 by Friedman’s test. For most activities the repeated measurements did not differ significantly (Table S2).

### Reported and observed HAQ in relation to grip force

Men had overall significantly higher grip force than women (Table 2).

As shown in Table 4, there was a significant negative correlation between grip force and most of the separate activities in HAQ2 in both women and men. Overall, the correlations between reported HAQ2 score and grip force were moderate, *r* = − 0.681, *p* < 0.001 in women and *r* = − 0.416, *p* < 0.003, in men.

**Table 4:** Correlations between grip force assessed with GRIPPIT mean right and left hands and the different activities in reported HAQ2, in women and men

	Women	Men
Grip force		
versus HAQ_1	− 0.510, ***p*** **< 0.001**	−0.362, ***p*** **= 0.011**
versus HAQ_2	−0.451, ***p*** **= 0.001**	− 0.344, ***p*** **= 0.015**
versus HAQ_3	−0.472, ***p*** **< 0.001**	− 0.340, ***p*** **= 0.017**
versus HAQ_4	−0.400, ***p*** **= 0.004**	− 0.331, ***p*** **= 0.020**
versus HAQ_5	−0.596, ***p*** **< 0.001**	− 0.337, ***p*** **= 0.018**
versus HAQ_6	−0.567, ***p*** **< 0.001**	− 0.414, ***p*** **= 0.003**
versus HAQ_7	−0.356, ***p*** **= 0.010**	− 0.277, *p* = 0.054
versus HAQ_8	−0.216, *p* = 0.129	− 0.366, ***p*** **= 0.010**
versus HAQ_9	−0.194, *p* = 0.172	− 0.349, ***p*** **= 0.014**
versus HAQ_10	−0.569, ***p*** **< 0.001**	− 0.414, ***p*** **= 0.003**
versus HAQ_11	−0.465, ***p*** **= 0.001**	− 0.218, *p* = 0.133
versus HAQ_12	−0.489, ***p*** **< 0.001**	− 0.365, ***p*** **= 0.010**
versus HAQ_13	−0.582, ***p*** **< 0.001**	− 0.379, ***p*** **= 0.007**
versus HAQ_14	−0.569, ***p*** **< 0.001**	− 0.371, ***p*** **= 0.009**
versus HAQ_15	−0.516, ***p*** **< 0.001**	− 0.414, ***p*** **= 0.003**
versus HAQ_16	−0.454, ***p*** **= 0.001**	− 0.364, ***p*** **= 0.010**
versus HAQ_17	−0.397, ***p*** **= 0.004**	−0.348, ***p*** **= 0.014**
versus HAQ_18	−0.585, ***p*** **< 0.001**	−0.367, ***p*** **= 0.010**
versus HAQ_19	−0.560, ***p*** **< 0.001**	−0.381, ***p*** **= 0.007**
versus HAQ_20	−0.466, ***p*** **= 0.001**	−0.376, ***p*** **= 0.008**
versus total HAQ2_score	−0.681, ***p*** **< 0.001**	−0.416, ***p*** **= 0.003**

Bold *p*-values are significant

*HAQ* Health Assessment Questionnaire

Looking at grip force and observed HAQ we found similar correlations, *r* = − 0.698, *p* < 0.001, in women and *r* = − 0.516, *p* < 0.001, in men.

### Reported and observed HAQ in relation to SOFI

The three parts of SOFI were similar in women and men (Table 2). The correlations between SOFI and the different questions in reported HAQ2 were calculated separately for women and men (Table 5).

**Table 5:** Correlations between SOFI hands, arms and legs and the different activities in reported HAQ2, in women and men

	Women			Men		
	SOFI hands	SOFI arms	SOFI legs	SOFI hands	SOFI arms	SOFI legs
HAQ_1	0.208, *p* = 0.144	0.097, *p* = 0.500	0.439, ***p*** **= 0.001**	0.076, *p* = 0.603	0.381, *p* = **0.007**	0.607, ***p*** **< 0.001**
HAQ_2	0.463, ***p*** **= 0.001**	0.313, *p* = **0.026**	0.349, ***p*** **= 0.012**	0.321, ***p*** **= 0.025**	0.341, *p* = **0.017**	0.627, ***p*** **< 0.001**
HAQ_3	0.291, ***p*** **= 0.038**	0.330, *p* = **0.018**	0.512, ***p*** **< 0.001**	0.161, *p* = 0.268	0.226, *p* = 0.118	0.652, ***p*** **< 0.001**
HAQ_4	0.313, ***p*** **= 0.026**	0.183 *p* = 0.198	0.429, ***p*** **= 0.002**	0.141, *p* = 0.333	0.248, *p* = 0.086	0.460, ***p*** **= 0.001**
HAQ_5	0.490, ***p*** **< 0.001**	0.175, *p* = 0.221	0.385, ***p*** **= 0.005**	0.089, *p* = 0.545	0.271, *p* = 0.060	0.546, ***p*** **< 0.001**
HAQ_6	0.447, ***p*** **= 0.001**	0.051, *p* = 0.722	0.485, ***p*** **< 0.001**	0.035, *p* = 0.813	0.111, *p* = 0.449	0.316, ***p*** **= 0.029**
HAQ_7	0.630, ***p*** **< 0.001**	0.301, ***p*** **= 0.032**	0.397, ***p*** **= 0.004**	0.252, *p* = 0.081	0.054, *p* = 0.713	0.417, ***p*** **= 0.003**
HAQ_8	0.320, ***p*** **= 0.022**	0.186, *p* = 0.190	0.643, ***p*** **< 0.001**	0.191, *p* = 0.189	0.248, *p* = 0.086	0.483, ***p*** **= 0.001**
HAQ_9	0.319, ***p*** **= 0.023**	0.094, *p* = 0.513	0.399, ***p*** **= 0.004**	0.030, *p* = 0.838	0.224, *p* = 0.122	0.579, ***p*** **< 0.001**
HAQ_10	0.483, ***p*** **< 0.001**	0.364, ***p*** **= 0.009**	0.454, ***p*** **= 0.001**	0.370, ***p*** **= 0.009**	0.419, ***p*** **= 0.003**	0.597, ***p*** **< 0.001**
HAQ_11	0.448, ***p*** **= 0.001**	0.271, p = 0.054	0.365, ***p*** **= 0.008**	0.054, *p* = 0.712	0.209, *p* = 0.152	0.531, ***p*** **< 0.001**
HAQ_12	0.458, ***p*** **= 0.001**	0.153, *p* = 0.284	0.318, ***p*** **= 0.023**	0.275, *p* = 0.056	0.326, ***p*** **= 0.022**	0.531, ***p*** **< 0.001**
HAQ_13	0.498, ***p*** **< 0.001**	0.302, ***p*** **= 0.031**	0.324, ***p*** **= 0.020**	0.223, *p* = 0.123	0.143, *p* = 0.328	0.356, ***p*** **= 0.013**
HAQ_14	0.585, ***p*** **< 0.001**	0.431, ***p*** **= 0.002**	0.510, ***p*** **< 0.001**	0.174, *p* = 0.240	0.305, ***p*** **= 0.033**	0.532, ***p*** **< 0.001**
HAQ_15	0.339, ***p*** **= 0.015**	0.193, *p* = 0.175	0.384, ***p*** **= 0.005**	0.237, *p* = 0.101	0.070, *p* = 0.635	0.349, ***p*** **= 0.015**
HAQ_16	0.463, ***p*** **< 0.001**	0.090, *p* = 0.530	0.241, *p* = 0.089	0.145, *p* = 0.319	0.067, *p* = 0.648	0.464, ***p*** **= 0.001**
HAQ_17	0.528, ***p*** **< 0.001**	0.078, *p* = 0.584	0.384, ***p*** **= 0.005**	0.189, *p* = 0.194	0.169, *p* = 0.246	0.525, ***p*** **< 0.001**
HAQ_18	0.368, ***p*** **= 0.008**	0.362, ***p*** **= 0.009**	0.505, ***p*** **< 0.001**	0.134, *p* = 0.358	0.340, ***p*** **= 0.017**	0.505, ***p*** **< 0.001**
HAQ_19	0.459, ***p*** **= 0.001**	0.232, *p* = 0.099	0.363, ***p*** **= 0.009**	0.035, *p* = 0.813	0.227, *p* = 0.177	0.434, ***p*** **= 0.002**
HAQ_20	0.434, ***p*** **= 0.001**	0.251, *p* = 0.076	0.579, ***p*** **< 0.001**	0.238, *p* = 0.099	0.119, *p* = 0.417	0.568, ***p*** **< 0.001**
HAQ_score	0.584, ***p*** **< 0.001**	0.316, ***p*** **= 0.024**	0.614, ***p*** **< 0.001**	0.367, ***p*** **= 0.009**	0.354, ***p*** **= 0.013**	0.728, ***p*** **< 0.001**

Bold *p*-values are significant

*SOFI* Signals of Functional Impairment, *HAQ* Health Assessment Questionnaire

In women, significant correlations were found both between SOFI hands and legs and most questions in HAQ2. SOFI arms were significantly correlated to the domains of grooming, rising, eating, reaching and other activities (questions 2, 3, 7, 10, 13, 14 and 18).

In men, SOFI hands did significantly correlate with only a few HAQ2 activities. Notably, SOFI legs but not SOFI hands correlated well with HAQ 16 and 17, although both questions are almost entirely dependent on hand function. SOFI arms correlated moderately with the questions about dressing, hygiene, reaching and other activities (questions 1, 2, 10, 12, 14 and 18). SOFI legs correlated significantly with all items (Table 5). In both women and men, the associations of observed HAQ with SOFI components were similar to those of HAQ2 with SOFI components.

Overall, the correlations between reported HAQ2 total score and SOFI index were moderate, *r* = 0.721, *p* < 0.001 in women, and *r* = 0.634, *p* < 0.001 in men. Similar moderate correlations were found between HAQ observed total score and SOFI index, *r* = 0.762, *p* < 0.001 in women and *r* = 0.693, *p* < 0.001 in men.

## Discussion

This study shows that women and men with established RA and low disease activity reported their activity limitations, assessed by the self-administered HAQ, in relatively good agreement with that noticed by an independent observer. However, in some activities the women as well as the men reported their abilities to be better than those observed, both before the observed performance and in the subsequent reported questionnaires. In both genders there was a negative correlation between grip force and most of the separate activities in HAQ. Furthermore, the functional impairment assessed by SOFI showed in women correlations especially between SOFI hands and legs and HAQ and in men mainly between SOFI legs and HAQ.

The HAQ disability index, specifically designed for arthritic patients, was among the first instruments to assess the patient’s own opinion of her/his health status. The Swedish version of HAQ, presented in 1988, showed a high degree of reliability and validity in patients with RA [20]. Neither in the original HAQ from Stanford [25] nor in the Swedish version [20], the validity was tested separately in women and men, but in these studies as well as in the present study, a strong correlation was found between the patient’s self-administered abilities and the observer’s opinion. In the US patients the correlation was *r* = 0.88 and in the Swedish patients *r* = 0.71. Of interest, in the latter report the authors suggested that men may tend to underreport daily life difficulties [20].

The agreement between patient reported HAQ and observed HAQ was, however, not true for all activities. As discrepancies were present also in the reported HAQ after the practical performance of the activities, we consider that the patient’s opinion was not dependent on unawareness of his/her own ability. Instead, the discrepancies may relate to divergent perceptions of disability. When, in the present study, the reported and observed activities differed, the patient’s assessment score was always lower than that observed, in both genders. As the patients relate to their own situation, their lower rating probably reflects that they after several years with RA may have changed their perception of what is difficult. This interpretation agrees with an earlier study [11] comparing the reported HAQ of 51 patients with that observed, in which the average difference was low, mean (SD) 0.09 (0.39), but wide in range and without any significant gender difference. In that study the patients’ over-reported functional ability was associated with longer disease duration, why they may have been more inclined to adapt to the loss of functional ability.

Due to the subjective nature of the observed HAQ, aspects of trustworthiness were considered regarding the data collection procedure [26]. The standardized protocol was developed and used to ensure consistent assessments based on the same criteria, and the different levels of difficulty were tested before they were applied to this study. To eliminate sources of bias, the time factor was not considered as an assessment criterion. Thus, the standardized observed assessment procedure estimated the functional performance regardless of the time factor. In addition, the patients did not receive any feedback from the observer on how their performances were graded in order not to affect the participants’ own subsequent assessments. To further avoid bias the observer was blinded for the result of the reported HAQ.

As HAQ reflects patient perceived abilities in performance limited to certain areas of daily life, many related to housework, one might suspect that men are not always aware of their abilities. However, this suspicion could not be verified here. Neither did the questions deal with rare activities as there were very few questions, which were not answered by the patients. We are thus confident that HAQ is a reliable instrument to assess activity limitation but realise the wish to add modern lifestyle activities [27], as well as supplements with the patient’s opinion about the impact of the disability [28].

Similar to our findings, many studies report higher HAQ scores in women than in men [2, 5, 6], mostly related to higher disease activity in women. However, in one study the HAQ score did not differ between genders at diagnosis [7]. Interestingly, in a study establishing normative values for HAQ scores in the general population in which the functional disability increased with age, the gender difference disappeared after adjustment for age [29].

Apart from disease activity and joint damage, grip force has been reported to correlate with HAQ score in patients with RA, women as well as men [10]. Also, in the present study we found a negative correlation between grip force and most of the separate activity scores in HAQ in both genders. To note, such a correlation was as well found in a few activities not obviously dependent on the use of hands, i.e. walking (in men). We suggest this to be explained by good muscle strength in hands and legs.

We could verify that the women with RA had significantly lower grip force than the men, with mean values close to those earlier reported after a disease duration of 5 years and lower than those of healthy referents [30]. Despite the discrepancy in grip force, the total HAQ score was not significantly different between genders. This finding resembles the situation in healthy persons, where women and men had similar HAQ score, despite lower grip force in the women [30]. Therefore, only grip force reduced to a certain level seems to have functional importance, which has been shown by grouping RA patients with respect to grip force [10].

Physical impairment, assessed by SOFI, also correlated with different activities in HAQ, but unequally between genders. In women, SOFI hands and legs correlated with most activities, whereas in men SOFI legs had the greatest impact on the HAQ activities. The association between impairment and activity limitation was, though, moderate which is in line with earlier reports [31, 32]. It is obvious that the range of movements of individual joints has different implications for the various activities of the HAQ, in agreement with a previous report [14] and that SOFI leg is an important predictor of HAQ [30].

This study has certain limitations. The study includes relatively small numbers of patients and these had relatively well-controlled disease. The interpretation of the present findings is thus restricted to patients with low disease activity and limited disability. It should also be mentioned that since a main aim of this study was to consider possible gender differences in HAQ, sampling was stratified by gender to avoid getting men underrepresented in the study. A strength is that the present study, unlike most earlier studies in this field, was performed during a period when active therapy with modern treatments had been instituted. A further strength is that all observed assessments were performed by the same observer in the same environment.

## Conclusions

In both women and men with RA and low disease activity the correlations between reported and observed HAQ scores were strong. We found no evidence supporting the possibility that the patient’s opinion was dependent on unawareness of his/her own ability. Instead, we suggest that when the patients scored their ability to be better than that observed it might reflect that the patients after several years with RA may have changed their opinion about what is difficult. Furthermore, activity limitation was closely related to grip force and to some extent to physical function in both women and men.

## Supplementary Information

**Additional file 1 **: **Table S1**. Reported HAQ2 and observed HAQ in women and men. The scores for the 17 questions are compared as well as the total HAQ score for all 7 domains. **Table S2**. Comparison of HAQ1, HAQ2 and HAQ3 in women and men respectively.

## Data Availability

The datasets used are available from the corresponding author on reasonable request.

## Abbreviations

bDMARDs: Biological disease-modifying anti-rheumatic drugs
cDMARDs: Conventional disease-modifying anti-rheumatic drugs
DAS28: Disease Activity Score calculated on 28 joints
HAQ: Health Assessment Questionnaire
RA: Rheumatoid arthritis
IQR: Interquartile range
SOFI: Signals Of Functional Impairment
