# Supplementary material for: Reported disability in relation to observed activity limitation, grip strength and physical function in women and men with rheumatoid arthritis
Source: BMC Rheumatol. 2021 May 3;5:13. doi: 10.1186/s41927-021-00184-5 (PMC8091772; doi:10.1186/s41927-021-00184-5)
Supplement: Supplementary file 1 — Additional file 1 : Table S1. Reported HAQ2 and observed HAQ in women and men. The scores for the 17 questions are compared as well as the total HAQ score for all 7 domains. Table S2. Comparison of HAQ1, HAQ2 and HAQ3 in women and men respectively. [file 41927_2021_184_MOESM1_ESM.docx]

Additional file

Table S1. Reported HAQ2 and observed HAQ in women and men. The scores for the 17 questions are compared as well as the total HAQ score for all 7 domains.

|  | Women | | | Men | | |
| --- | --- | --- | --- | --- | --- | --- |
|  | HAQ2 reported | HAQ observed | p-value | HAQ_2 reported | HAQ observed | p-value |
| HAQ_3 | 0.0 (0.0) | 0.0 (0.0) | 1.0 | 0.0 (0.0) | 0.0 (1.0) | 0.564 |
| HAQ_4 | 0.0 (0.0) | 0.0 (1.0) | 0.180 | 0.0 (0.0) | 0.0 (0.0) | 0.705 |
| HAQ_5 | 0.5 (1.0) | 0.0 (1.0) | 0.317 | 0.0 (0.0) | 0.0 (1.0) | 0.059 |
| HAQ_6 | 0.0 (1.0) | 0.0 (1.0) | **0.021** | 0.0 (0.0) | 0.0 (1.0) | **0.021** |
| HAQ_7 | 0.0 (0.0) | 0.0 (0.0) | 1.0 | 0.0 (0.0) | 0.0 (0.0) | 0.257 |
| HAQ_8 | 0.0 (1.0) | 0.0 (1.0) | **0.005** | 0.0 (0.0) | 0.0 (0.8) | **0.004** |
| HAQ_9 | 0.0 (1.0) | 0.0 (1.0) | 0.102 | 0.0 (0.0) | 0.0 (1.0) | 0.083 |
| HAQ_10 | 0.0 (3.0) | 0.0 (2.0) | **0.008** | 0.0 (1.0) | 0.5 (1.8) | 0.097 |
| HAQ_11 | 0.0 (0.0) | 0.0 (0.0) | 0.157 | 0.0 (0.0) | 0.0 (1.0) | 0.317 |
| HAQ_12 | 0.0 (1.0) | 0.0 (1.0) | 0.035 | 0.0 (1.0) | 1.0 (1.0) | <0.001 |
| HAQ_13 | 1.0 (1.0) | 1.0 (1.0) | 0.132 | 0.0 (1.0) | 0.0 (1.0) | 0.034 |
| HAQ_14 | 0.0 (0.0) | 0.0 (0.5) | 0.180 | 0.0 (0.0) | 0.0 (1.0) | 0.083 |
| HAQ_15 | 0.0 (0.0) | 0.0 (0.0) | 1.0 | 0.0 (0.0) | 0.0 (0.0) | 1.0 |
| HAQ_16 | 0.0 (1.0) | 0.0 (1.0) | 0.248 | 0.0 (0.8) | 0.0 (1.0) | **0.021** |
| HAQ_17 | 0.0 (1.0) | 0.0 (0.5) | 0.157 | 0.0 (0.0) | 0.0 (0.0) | 0.083 |
| HAQ_18 | 0.0 (1.0) | 0.0 (1.0) | **0.025** | 0.0 (0.0) | 0.0 (1.0) | **0.006** |
| HAQ_20 | 0.0 (1.0) | 0.0 (1.0) | 0.102 | 0.0 (1.0) | 0.0 (1.0) | 0.564 |
| HAQ_score | 0.43 (0.96) | 0.57 (0.86) | **0.002** | 0.21 (0.57) | 0.43 (0.96) | **<0.001** |

Values are medians (IQR); p-values are differences between reported HAQ2 and observed HAQ in respective gender. Bold p-values are significant. HAQ, Health Assessment Questionnaire.

Table S2. Comparison of HAQ1, HAQ2 and HAQ3 in women and men respectively.

|  | Women | | | | Men | | | |
| --- | --- | --- | --- | --- | --- | --- | --- | --- |
|  | HAQ1 | HAQ2 | HAQ3 | p-value* | HAQ1 | HAQ2 | HAQ3 | p-value* |
| HAQ_1 | 0.0 (0.0) | 0.0 (0.0) | 0.0 (0.0) | 0.895 | 0.0 (0.0) | 0.0 (0.0) | 0.0 (0.0) | 0.097 |
| HAQ_2 | 0.0 (1.0) | 0.0 (1.0) | 0.0 (1.0) | **0.045** | 1.0 (1.0) | 0.0 (1.0) | 0.0 (1.0) | 0.558 |
| HAQ_3 | 0.0 (0.8) | 0.0 (0.0) | 0.0 (0.0) | **0.039** | 0.0 (1.0) | 0.0 (0.0) | 0.0 (1.0) | 0.311 |
| HAQ_4 | 0.0 (0.0) | 0.0 (0.0) | 0.0 (1.0) | 0.264 | 0.0 (1.0) | 0.0 (0.0) | 0.0 (1.0) | 0.273 |
| HAQ_5 | 0.0 (1.0) | 0.5 (1.0) | 0.0 (1.0) | 0.323 | 0.0 (0.0) | 0.0 (0.0) | 0.0 (0.0) | 0.846 |
| HAQ_6 | 0.0 (0.8) | 0.0 (1.0) | 0.0 (1.0) | 0.071 | 0.0 (0.0) | 0.0 (0.0) | 0.0 (0.0) | 0.311 |
| HAQ_7 | 0.0 (0.0) | 0.0 (0.0) | 0.0 (0.0) | 0.651 | 0.0 (0.0) | 0.0 (0.0) | 0.0 (0.0) | 0.247 |
| HAQ_8 | 0.0 (1.0) | 0.0 (1.0) | 0.0 (1.0) | 0.303 | 0.0 (0.0) | 0.0 (0.0) | 0.0 (0.0) | 0.197 |
| HAQ_9 | 0.0 (1.0) | 0.0 (1.0) | 0.0 (1.0) | 0.338 | 0.0 (0.0) | 0.0 (0.0) | 0.0 (0.0) | 0.819 |
| HAQ_10 | 0.0 (1.0) | 0.0 (3.0) | 0.0 (2.0) | 0.920 | 0.0 (1.0) | 0.0 (1.0) | 0.0 (1.0) | 0.913 |
| HAQ_11 | 0.0 (0.0) | 0.0 (0.0) | 0.0 (0.0) | 0.236 | 0.0 (0.0) | 0.0 (0.0) | 0.0 (0.5) | 0.819 |
| HAQ_12 | 0.0 (1.0) | 0.0 (1.0) | 0.0 (1.0) | 0.174 | 0.0 (1.0) | 0.0 (1.0) | 0.0 (1.0) | 0.607 |
| HAQ_13 | 1.0 (1.0) | 1.0 (1.0) | 1.0 (1.0) | 0.094 | 0.0 (1.0) | 0.0 (1.0) | 0.0 (1.0) | 0.846 |
| HAQ_14 | 0.0 (0.0) | 0.0 (0.0) | 0.0 (0.0) | 0.368 | 0.0 (1.0) | 0.0 (0.0) | 0.0 (0.0) | 0.135 |
| HAQ_15 | 0.0 (0.0) | 0.0 (0.0) | 0.0 (0.0) | **0.030** | 0.0 (0.0) | 0.0 (0.0) | 0.0 (0.0) | 0.607 |
| HAQ_16 | 0.0 (1.0) | 0.0 (1.0) | 0.0 (1.0) | **0.029** | 0.0 (1.0) | 0.0 (0.8) | 0.0 (0.0) | 0.405 |
| HAQ_17 | 0.0 (1.0) | 0.0 (1.0) | 0.0 (1.0) | 0.417 | 0.0 (0.0) | 0.0 (0.0) | 0.0 (0.0) | 0.607 |
| HAQ_18 | 0.0 (1.0) | 0.0 (1.0) | 0.0 (1.0) | 0.973 | 0.0 (0.0) | 0.0 (0.0) | 0.0 (0.0) | 0.472 |
| HAQ_19 | 0.0 (1.0) | 0.0 (1.0) | 0.0 (1.0) | 0.368 | 0.0 (0.0) | 0.0 (0.0) | 0.0 (0.0) | 1.0 |
| HAQ_20 | 0.0 (1.0) | 0.0 (1.0) | 0.0 (1.0) | 0.338 | 0.0 (1.0) | 0.0 (1.0) | 0.0 (0.0) | 0.069 |
| HAQ score | 0.50 (0.9) | 0.50 (0.9) | 0.38 (0.9) | **0.033** | 0.25 (0.8) | 0.13 (0.8) | 0.13 (0.8) | 0.542 |

Values are medians (IQR). P-values are differences between HAQ1, HAQ2 and HAQ3 in respective gender by Friedman’s test. Bold values are significant. HAQ, Health Assessment Questionnaire.
